# The True Position of the Specialist

**Published:** 1907-12-14

**Authors:** 


### The Hospital

A Journal of

The Medical Sciences and Hospital Administration.

New Series. No. 38, Vol. II. [No. 1110, Vol. XLIII.] SATURDAY, DECEMBER 14, 1907.

### THE TRUE POSITION OF THE SPECIALIST.

Specialism in medical practice, whether considered as a name or as a custom, has in its time been subjected to much criticism, and even to a considerable amount of denunciation. By the general public, however, it has been exalted to a high professional level, and it continues to receive many tokens of popular confidence and regard. Under the same influence, medical practice has been divided and subdivided into a number of definite and sharply-limited areas, and over each of these it is fondly imagined an appropriate group of specialists skilfully presides. In cases of doubt or difficulty there thus readily arises, from the patient or his friends, a ready appeal for the verdict of one or other of these supposed authorities, the choice of the individual adviser being determined by his alleged relation to the diseases of the organ in which, in the case in question, the fault is presumed to lie. In many instances, indeed, there is the plain suggestion that the family practitioner is in complete ignorance of pathological conditions affecting particular parts of the body, and that to ask his advice in reference to these is both useless and unnecessary. Hence not infrequently the patient places himself directly under the care of an adviser who, on more or less valid grounds, is reputed to be a specialist in the department of practice from which help is needed. Opposed to all this is the view of not a few medical men that so-called specialism is associated with much pretence and even with some degree of quackery; that it leads to many unnecessary operative measures; and that under it patients who might suitably be in the care of a general practitioner are drawn into the net of the practising specialist. This latter view, it may be urged, is an extreme doctrine, and is no more entitled to respect than is the worship of specialism so extensively practised by the general public. Yet it exists, and signs are not wanting that it may ere long form a motive for action on the part of a not inconsiderable section of the profession. For this reason, and also for the sake of finding a safe basis and wise limit for what is beyond question a widespread custom, it would be well that the practice of specialism should receive an authoritative examination from those whose duty it is to endeavour to guide professional opinion.

Perhaps the most commanding aspect of specialism is the extent to which it has developed during comparatively recent years. And it is a safe economic maxim that a custom which displays vitality and attracts confidence contains at least some element of value and utility. Evil may perchance be mingled with the good, but where there is life and growth, capacity and service cannot be wholly absent. Indeed, it must be accepted that specialism in medicine, as in other departments of human activity, is an inevitable outgrowth of the advance of knowledge, and that an endeavour to suppress it is a vain and foolish dream. One of its most secure defences is the knowledge of disease and of treatment which has been acquired by its practice, and at the present day has passed into the common stock of professional ability to relieve and reduce the sum of human suffering. It is entirely beyond question, that, allowing a due discount for all exaggerated or unfounded claims, many of the most valuable additions to the equipment of the practitioner are the outcome of observations made in the first instance by those engaged in special branches of practice. As is so common in human affairs, these undoubted gains may be associated with possible or actual losses. But the reputation of the profession, not less than the interests of humanity, demand that the advantages must be conserved at all costs, though this by no means implies that abuses are to be suffered without protest. In short, it will command fairly general recognition to say that specialism, in some measure at least, must be admitted to be a necessity, and that what is desired is not its suppression but its correct adjustment in the scheme of professional responsibility.

To secure this true perspective for specialism, it is essential to recognise that its immediate concern is with local ailments and disturbances. Inevitably, therefore, the specialist thinks mainly of the organ upon which his attention is concentrated, and is apt to lose sight of the patient considered as a harmonious physiological entity. Hence, both his diagnosis and treatment must be inclined to be based upon a restricted view, and to have regard to local conditions rather than to the wider aspects of the case. It is the appreciation of these facts which is essential to the definition of the position of the specialist in relation to the general ethical claim of the profession.

There may be, probably there are, abuses which depend on the perverted judgment, and sometimes perhaps, on the moral obliquity, of individual practitioners engaged in various departments of special practice. These, so far as they are open to redress, must be left to the natural justice of time and the event. But the essential evil of specialism on which a general professional judgment may be pronounced, arises when a local lesion is treated without a confident knowledge of the general clinical condition of the patient. In other words, the specialist who practises in this manner is to be condemned as one acting contrary to the claims both of science and of ethics. It therefore appears that specialism is only in its true position when it can afford special knowledge or dexterity as a supplement to the general examination of the patient. So defined, its function is one of high service. But, divorced from a complete clinical investigation, specialism is bad in principle and dangerous in practice. To treat, for example, a case of iritis, of optic atrophy, or of laryngeal paralysis without such an investigation, is unjust to the patient and false to the claims of professional duty. In short, special practice, to be capable of defence, must be associated with, and not detached from, the sound and thorough methods of general medicine.

